# The early bird in renal rescue: timing matters in acute kidney injury management—insights from target trial emulation

**DOI:** 10.3389/fmed.2025.1645046

**Published:** 2025-09-04

**Authors:** Miaowen She, Jinhu Zhuang, Xi Chen, Lili Hu, Thuan-Quoc Thach, Kin Cheung, Xiaxia Yu, Mengyi Wang, Xiang Zheng, Yong Liu

**Affiliations:** ^1^Department of Critical Care Medicine, Taihe Hospital, Hubei University of Medicine, Shiyan, Hubei, China; ^2^Shenzhen Health Development Research and Data Management Center, Shenzhen, China; ^3^Department of Pharmacy, Shenzhen Hospital, Southern Medical University, Shenzhen, China; ^4^Intensive Care Unit, Shenzhen Hospital, Southern Medical University, Shenzhen, Guangdong, China; ^5^Department of Psychiatry, Li Ka Shing Faculty of Medicine, The University of Hong Kong, Hong Kong, China; ^6^School of Nursing, The Hong Kong Polytechnic University, Hong Kong, China; ^7^School of Biomedical Engineering, Shenzhen University Medical School, Shenzhen University, Shenzhen, China

**Keywords:** acute kidney injury, renal replacement therapy, timing, mortality, target trial emulation

## Abstract

**Background:**

Acute kidney injury (AKI) is prevalent in critically ill patients. The optimal timing for initiating renal replacement therapy (RRT) remains unsettled. Clinical intuition suggests early initiation could be beneficial, but evidence from studies is inconsistent.

**Methods:**

A target trial emulation was conducted using the Medical Information Mart for Intensive Care-IV (MIMIC-IV) database. Two cohorts were analyzed: broader cohort (stage ≥1, *N* = 7,607) and severe cohort (stage 3, *N* = 943). Cox proportional hazards models with inverse probability weighting (IPW) were used to estimate the causal effect on 90-day and 30-day mortality, with sensitivity analyses using accelerated failure time models and augmented inverse probability weighting.

**Results:**

In the broader cohort, early initiation was associated with lower 90-day (hazard ratio (HR): 0.653, 95% confidence interval (CI): 0.512–0.834) and 30-day mortality (HR 0.649, 95% CI 0.504–0.835). However, accelerated failure time models indicated no survival benefit and potentially worse outcomes, with a 14.8% reduction in 90-day survival and a 14.1% reduction in 30-day survival. Augmented inverse probability weighting (AIPW) analysis further confirmed these findings, showing a risk difference of 2.6 percentage points between early and late initiation of RRT. In the severe cohort, early initiation was associated with lower 90-day mortality (HR: 0.561, 95% CI: 0.341–0.921) and 30-day mortality (HR: 0.604, 95% CI: 0.357–1.022), with accelerated failure time models confirming longer survival. Augmented inverse probability weighting analysis in this group showed a risk difference of 1.7 percentage points.

**Conclusion:**

Early initiation appears beneficial in the severe cohort (stage 3), but not in the broader cohort (stage ≥1), where it could even be harmful. This highlights the need for personalized treatment based on the severity of acute kidney injury and further research to optimize the timing of renal replacement therapy.

## 1 Introduction

Epidemiological studies over the past few decades have shown that the incidence of acute kidney injury (AKI) in critically ill patients can be as high as 61.3%, with mortality rates exceeding 50% (1). The high incidence and mortality figures associated with AKI highlight it as a critical issue that needs to be urgently addressed in the intensive care unit (ICU). Effective management can significantly reduce the risk of patient mortality and improve outcomes (2). Renal replacement therapy (RRT) is a key intervention for treating AKI, and the timing of its initiation directly impacts patient outcomes (3). Early studies on RRT primarily focused on the techniques and methods of performing RRT, with less emphasis on the optimal timing of RRT initiation (4). In recent years, there has been a notable advancement in our comprehension of the pathophysiological mechanisms underlying AKI, and researchers have shifted their focus to assessing the impact of the timing of RRT initiation on patient prognosis (5).

There is indirect evidence to suggest that early RRT could confer a survival benefit (6, 7). Nevertheless, two observational studies have indicated that patients with delayed initiation of RRT had lower 90-day mortality (8, 9). Furthermore, another study indicated that very early RRT therapy in patients with sepsis was associated with adverse outcomes (10). Early RRT could result in benefits in terms of improved control of fluid and electrolyte balance, removal of uremic toxins, and a reduction in the incidence of complications such as gastric hemorrhage and metabolic encephalopathy (11). Intuitively, delaying the initiation of RRT does not seem to show any immediate benefit; however, it could allow the stabilization of a patient’s condition before RRT is commenced, thereby potentially avoiding the need for such risky support (12).

At the same time, multiple clinical trials have assessed the influence of the timing of RRT initiation on the prognosis of patients with AKI. For example, both the AKIKI (Artificial Kidney Initiation in Kidney Injury) study and the STARRT-AKI (Standard Timing of Initiation of Renal Replacement Therapy in Acute Kidney Injury) trial did not show that early initiation of RRT improves survival rates (6, 9). These findings suggest that, in the absence of life-threatening complications, delaying the initiation of RRT may not negatively affect patient survival rates. Moreover, the IDEAL-ICU study revealed that there was no statistically significant difference in 90-day mortality between patients who underwent early RRT and those who underwent delayed RRT for severe AKI (10). However, these study results are controversial. The ELAIN (Early vs. Late Initiation of Renal Replacement Therapy in Acute Kidney Injury) trial showed that, in AKI patients, early initiation of RRT compared to delayed initiation resulted in a reduction in all-cause mortality at 90 days (13).

Randomized controlled trials (RCTs) are regarded as the gold standard for evaluating the effects of medical interventions, but they have inherent limitations (14, 15). First, although recent RCTs, including the AKIKI and STARRT-AKI studies, have included elderly patients, they still exclude those with advanced comorbidities, such as metastatic cancer and irreversible organ failure, and those requiring palliative care (16). This gap is addressed by examining real-world clinical cohorts in this study through the inclusion of these underrepresented, high-risk populations. Second, to reduce variability and enhance reliability, RCTs are typically conducted in tightly controlled environments that could differ from real-world clinical settings, thereby limiting the external validity of their findings (17). Furthermore, RCTs face challenges in analyzing time-varying factors, such as dynamic treatment adjustments and evolving comorbidities, which are critical determinants of treatment responses in real-world practice (16, 18). Lastly, RCTs can be costly and time-consuming, potentially delaying the rapid translation of emerging therapies into clinical use. Consequently, RCT findings may not fully reflect patient populations in real-world settings, particularly in dynamic clinical contexts such as intensive care units, where patient conditions are highly complex and rapidly evolving (15).

Despite recent RCTs not supporting early RRT initiation, observational data indicate that >50% of clinicians initiate RRT early during shock or respiratory failure due to perceived urgency (10, 19). There is an urgent need to clarify whether the RCT designs are unable to identify the true effects of RRT or if this is a matter of clinicians’ expectations and desire to care for patients (5).

In contrast to conventional observational studies, this study adopts the target trial emulation framework (20, 21), which rigorously defines treatment strategies, specifically distinguishing between early and delayed RRT initiation. The framework also emulates dynamic treatment allocation through the cloning method and resolves time-varying confounding via doubly robust estimation using the augmented inverse probability weighting (AIPW). We further employed inverse probability weighted Cox proportional hazards models and accelerated failure time (AFT) models to address potential survival analysis biases (22, 23). Traditional observational studies often rely on Cox regression or propensity score matching, which inadequately address immortal time bias and time-varying confounding. For instance, Hernán (20) demonstrated that conventional approaches erroneously incorporate post-treatment covariates into models, thereby introducing bias. By emulating the intention-to-treat principle of target trials through a cloning-censoring-weighting structure, this study integrates both propensity score and outcome models with AIPW, thereby producing more reliable estimates of causal effect.

## 2 Methods

### 2.1 Data source

This retrospective analysis leveraged the publicly available Medical Information Mart for Intensive Care-IV (MIMIC-IV) database, which contains de-identified electronic health records of patients admitted to critical care units at Beth Israel Deaconess Medical Center (BIDMC) between 2008 and 2019. The dataset has been reviewed and approved by the Institutional Review Boards of the Massachusetts Institute of Technology (MIT) and BIDMC. Owing to its large, heterogeneous cohort and wide spectrum of ICU stay types, MIMIC-IV facilitates the broad generalizability of the findings (24, 25).

### 2.2 Eligibility criteria for the emulated trial

#### 2.2.1 Target trial 1: exploratory analysis in broad AKI population

To comprehensively evaluate the potential impact of early RRT initiation across the AKI spectrum, we first conducted an exploratory target trial by enrolling patients admitted for the first time to a non-neurological ICU. These patients met the Kidney Disease: Improving Global Outcomes (KDIGO) criteria for AKI stage ≥1. Eligibility criteria included an ICU stay exceeding 72 h, respiratory rate >10 breaths per min, no prior chronic kidney disease, and full code status (26). Patients were required to have ≥12 h of hospitalization prior to randomization to ensure adequate covariate data collation, with complete baseline covariates available at treatment assignment. While the current guidelines lack consensus on RRT timing in early-stage AKI, this exploratory analysis aimed to generate hypotheses about potential benefits or risks of early intervention even in cases with mild renal impairment.

#### 2.2.2 Target trial 2: primary analysis in stage 3 AKI

Recognizing the limited clinical adoption of early RRT in mild AKI (Stage 1–2) and the inherent reversibility of many early cases, we focused our primary analysis on patients with KDIGO Stage 3 AKI at baseline, among whom decisions to initiate RRT carry greater clinical urgency and equipoise. This refined cohort addresses key limitations of Target Trial 1 by excluding patients unlikely to require RRT due to transient or self-limited AKI. This helps in prioritizing a clinically actionable population, where delayed versus early RRT strategies are actively debated, and reducing potential confounding from heterogeneous practice patterns in milder AKI stages.

Eligibility criteria mirrored Target Trial 1 except for the AKI severity requirement (Stage 3 vs. Stage ≥1). This staged analytical approach aligns with recent consensus statements advocating for severity-stratified trials in critical care nephrology, while maintaining methodological rigor through harmonized covariate adjustment and identical immortal time bias mitigation strategies across both trials (27, 28).

### 2.3 Target trial emulation

To estimate the effect of early initiation of RRT on mortality in patients with AKI without a prior history of RRT during the ICU admission, we conducted an emulation of a target trial comparing initiation of RRT within 1 h of AKI diagnosis versus delayed initiation (including patients with delayed RRT initiation or no RRT during follow-up). Patients were eligible for the target trial in the first hour they met the eligibility criteria and for each subsequent hour they met the eligibility criteria, up to 72 h (Supplementary Table 1). This period was chosen as most of the clinical timing of RRT initiation occurs during this period, and also to minimize heterogeneity between patients.

### 2.4 Primary and secondary outcomes

The primary outcome measure was 90-day all-cause mortality, chosen for its clinical significance in evaluating the long-term impact of RRT initiation strategies. The secondary outcome, 30-day mortality, was selected to assess short-term treatment efficacy.

### 2.5 Missing data

The missing values for key non-time-dependent variables, such as gender, were directly removed. For other non-critical time-independent variables, multiple imputation chained equations (MICE) were employed, using predictive mean matching to enhance the accuracy of imputation. Meanwhile, time-dependent clinical data, including blood pressure, heart rate, and scores, were imputed using the values from the preceding hour (see Supplementary materials) (29, 30).

### 2.6 Data processing

This study followed the analytical framework of the established MIMIC-IV dataset. As a publicly accessible compendium of critical care data, MIMIC-IV is derived from a single medical center and has obtained approval from the Institutional Review Boards of BIDMC in Boston, USA, and the MIT (24, 25). Given the de-identified nature of the data, the necessity for individual patient consent is waived. The database is a rich repository, capturing a spectrum of baseline covariate information encompassing patient demographics, extant comorbidities, initial vital signs, and baseline laboratory values. It further extends its scope by collecting time-varying covariate data through the systematic recording of vital signs and laboratory parameters within the ICU. Of particular note is the inclusion of hourly physiological data, meticulously validated by seasoned ICU nursing staff, which enhances the dataset’s reliability.

To construct the dataset for this study, Navicat Premium version 16 was used to connect to the MIMIC-IV database, and R version 4.3.2 software was employed for statistical analysis. All the R code is available at https://github.com/Shemiaowen/RRT_emulation.

### 2.7 Statistical analysis

#### 2.7.1 Cloning

To address the ambiguity in assigning individuals to treatment strategies during the grace period (72 h post-baseline), each eligible subject was cloned to create two identical replicates. This approach emulated a target trial design where patients could hypothetically follow different strategies under identical baseline conditions. Each clone was assigned to one of the two predefined strategies at time zero (T0): (1) initiating therapy within the grace period; (2) never initiating therapy. Clones violating their assigned strategy (initiating therapy after the grace period for clone 2) were censored at the time of deviation. To prevent bias from informative censoring, inverse probability weighting (IPW) was applied to adjust for time-varying confounders. Importantly, events occurring during the grace period (such as death) were assigned to all clones, ensuring unbiased allocation of outcomes. This method prioritizes estimation of the per-protocol effect (the effect of sustained adherence to strategies) rather than the intention-to-treat effect, as baseline assignment is non-unique due to cloning. The methodology aligns with emulation frameworks for dynamic treatment strategies in observational data (31).

#### 2.7.2 Censoring

The study implemented hourly checkpoints to assess the fidelity of the replicates to their assigned treatment strategy. Any deviation from the assigned strategy resulted in the censoring of the respective replicate. This process was critical in maintaining analytical consistency, as it emulated the strict protocol adherence characteristic of well-controlled clinical trials, ensuring that the analysis reflected the intended treatment effects without the confounding influence of protocol violations (32).

#### 2.7.3 Weighting

A time-varying weight was assigned to each individual to correct any potential selection bias that could have been introduced by the censoring process (33, 34). These weights were calculated based on the conditional probability that a replicate remains on its assigned treatment strategy, contingent upon the individual’s baseline characteristics. Logistic regression models were employed to calculate this weight, with the initiation of RRT treated as the dependent variable (Yes/No) and potential variables treated as the independent variables. The independent variables included the time interval since the fulfillment of the inclusion criteria, age, gender, and comorbidities as measured by the Charlson comorbidity index (CCI), sequential organ failure assessment (SOFA), heart rate, respiratory rate, diastolic blood pressure, oxygen saturation, presence of sepsis, and the use of vasoactive drugs (see Supplementary materials).

After estimating the propensity score, stable inverse probability weights (IPW) were calculated to adjust for confounders. Additionally, in this population, 90-day mortality was subsequently assessed using a weighted Cox model to determine the distribution from time to event. The model also included systolic blood pressure, mean arterial pressure, temperature, pH, bicarbonate, lactate, hemoglobin, blood urea nitrogen, and creatinine levels, as these values could have an independent effect on mortality regardless of the patient’s decision to start RRT (35, 36). Hazard ratios (HR) are presented as the mean treatment effect for the study. Survival curves were constructed using a stratified Cox model, and Cox models with IPW were applied to evaluate 90-day mortality, focusing on the timing of event occurrence (see Supplementary materials).

We calculated standardized mean differences (SMDs) for all baseline variables before and after weighting, to assess the balance of baseline covariates between treatment groups after inverse probability weighting (IPW). An absolute SMD < 0.1 was considered indicative of good balance between groups. This method was used to ensure that the weighting procedure effectively minimized potential confounding due to observed baseline differences.

### 2.8 Sensitivity analysis

The Cox model’s fundamental assumption that the hazard ratio remains constant over time requires validation through verification of the proportional hazards (PH) assumption. Despite our efforts to minimize bias using the IPW strategy, the test outcomes revealed that the constant PH assumption was not met. This suggested that the traditional Cox model may not be adequate for capturing the dynamic changes in the hazard ratio over time (37, 38). Accordingly, we further explored the AFT model as an alternative analytical approach. In contrast to the traditional Cox proportional hazards model, the AFT model does not rely on the constant PH assumption (39), thereby leading to a more flexible approach to model the relationship between survival time and covariates. This model controls for potential confounding factors through stratification, thereby providing a more precise estimate of the risk of death. Moreover, to address the issue of heteroscedasticity, we calculated robust standard errors within the model to more accurately reflect the variability of the estimates across samples. We established a two-tailed *p*-value threshold of 0.05.

To further ensure the robustness of the findings, we replicated the main analyses using the robust statistical method of AIPW to complement the findings (40).

To evaluate potential multicollinearity among covariates included in the IPW models, we calculated variance inflation factors (VIFs) for all baseline variables. A VIF > 5 was considered indicative of concerning multicollinearity. Variables with high VIF were examined and, if necessary, excluded or combined to ensure model stability.

## 3 Results

### 3.1 Descriptive statistics for the MIMIC-IV cohort

**Figure 1:**
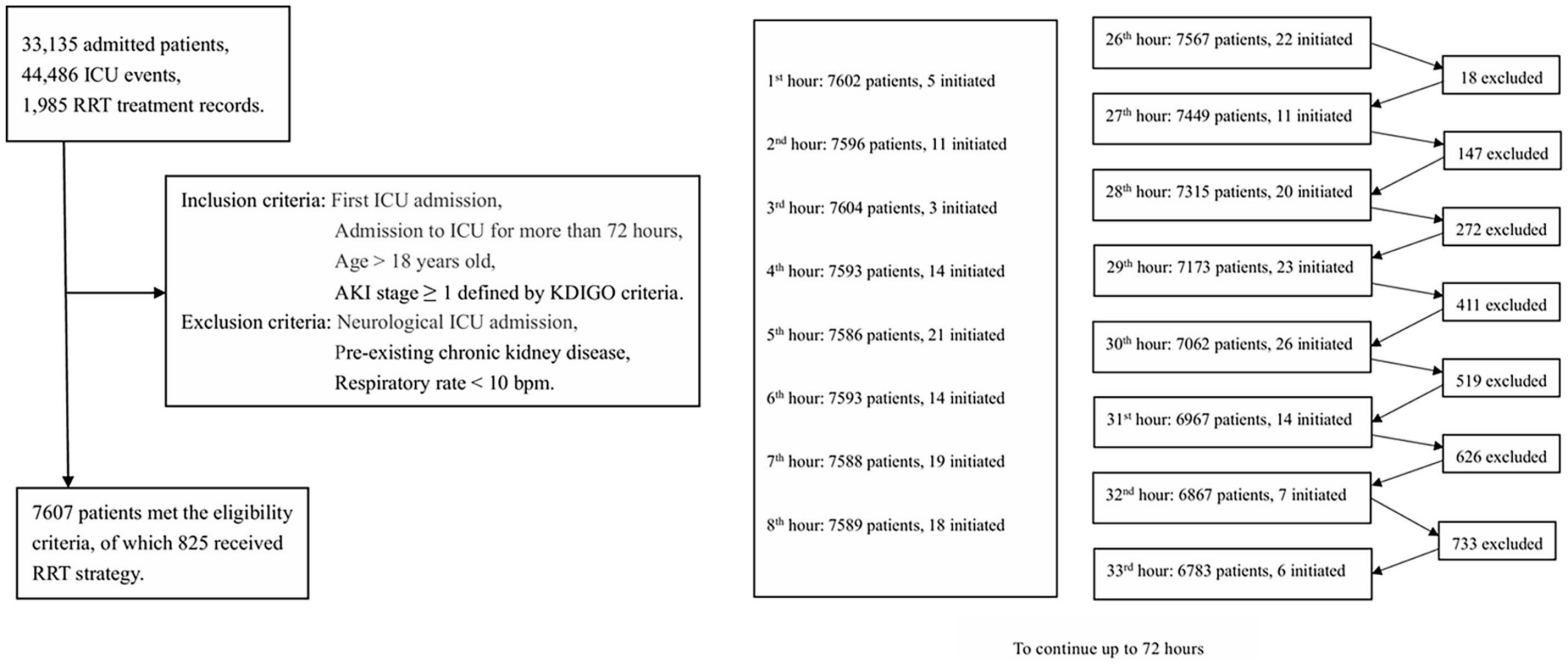
Study cohort.

A total of 7,607 patients met the inclusion criteria for target trial 1, with 825 receiving the RRT strategy within a 72-h window (refer to Figure 1, see Supplementary Table 2), while 943 patients met the inclusion criteria for target trial 2. The baseline characteristics for eligible patients are tabulated in Table 1.

**Table 1:** Baseline characteristics for target trial 1 and 2 eligible individuals (only information at hour 1 is selected).

Variable	Description	Target trial 1	Initiation RRT (*N* = 825)	*p*	Target trial 2	Initiation RRT (*N* = 206)	*p*
		No initiation RRT (*N* = 6,782)			No initiation RRT (*N* = 737)		
Age	Mean (SD)	66.33 (15.61)	62.51 (14.28)	<0.001	64.44 (15.33)	62.42 (13.75)	0.088
Female	*n* (%)	2,768 (40.8)	299 (36.2)	0.013	302 (41.0)	78 (37.9)	0.469
Race, *n* (%)	White	4,358 (64.3)	480 (58.2)	0.004	448 (60.8)	132 (64.1)	0.875
	Black	1,006 (14.8)	140 (17.0)		142 (19.3)	35 (17.0)	
	Asian	239 (3.5)	23 (2.8)		20 (2.7)	7 (3.4)	
	American Indian/Alaska Native	23 (0.3)	2 (0.2)		2 (0.3)	0 (0.0)	
	Hispanic/Latino	299 (4.4)	46 (5.6)		38 (5.2)	9 (4.4)	
	Others	857 (12.6)	134 (16.2)		87 (11.8)	23 (11.2)	
HR	Mean (SD)	87.52 (9.87)	87.04 (9.36)	0.183	86.44 (9.97)	87.14 (10.26)	0.374
RR	Mean (SD)	20.09 (3.17)	20.00 (3.20)	0.475	19.24 (2.96)	19.39 (2.88)	0.535
SBP (mmHg)	Mean (SD)	113.38 (13.91)	113.15 (13.84)	0.642	110.52 (13.26)	111.33 (13.12)	0.440
DBP (mmHg)	Mean (SD)	60.36 (8.63)	60.34 (8.68)	0.950	58.59 (8.29)	58.74 (8.29)	0.812
MBP (mmHg)	Mean (SD)	73.40 (9.43)	73.31 (9.49)	0.795	71.50 (9.05)	72.05 (9.04)	0.436
Temperature	Mean (SD)	36.87 (0.28)	36.88 (0.29)	0.080	36.77 (0.26)	36.78 (0.29)	0.839
SpO2	Mean (SD)	97.25 (1.70)	97.25 (1.61)	0.987	97.54 (1.60)	97.45 (1.70)	0.469
Mechanical ventilation, *n* (%)	No	4,250 (62.7)	366 (44.4)	<0.001	496 (67.3)	104 (50.5)	<0.001
	Yes	2,532 (37.3)	459 (55.6)		241 (32.7)	102 (49.5)	
Vasopressor, *n* (%)	No	6,651 (98.1)	792 (96.0)	<0.001	719 (97.6)	196 (95.1)	0.116
	Yes	131 (1.9)	33 (4.0)		18 (2.4)	10 (4.9)	
Sepsis, *n* (%)	No	2,696 (39.8)	194 (23.5)	<0.001	288 (39.1)	58 (28.2)	0.005
	Yes	4,086 (60.2)	631 (76.5)		449 (60.9)	148 (71.8)	
CCI	Mean (SD)	6.83 (3.00)	7.20 (2.92)	0.001	7.30 (3.13)	7.62 (2.96)	0.182
SOFA	Mean (SD)	7.32 (3.86)	11.98 (4.29)	<0.001	8.20 (3.62)	11.86 (4.13)	<0.001
SAPS II	Mean (SD)	43.08 (14.50)	54.39 (15.63)	<0.001	44.95 (14.72)	54.05 (15.68)	<0.001
GCS	Mean (SD)	14.92 (0.34)	14.90 (0.42)	0.151	14.87 (0.46)	14.89 (0.44)	0.589
pH	Mean (SD)	7.37 (0.06)	7.37 (0.06)	0.333	7.36 (0.06)	7.36 (0.07)	0.926
Bicarbonate (mmol/L)	Mean (SD)	22.67 (3.10)	22.55 (3.25)	0.282	21.75 (3.06)	21.85 (3.17)	0.669
Potassium (mmol/L)	Mean (SD)	4.21 (0.42)	4.23 (0.45)	0.162	4.30 (0.48)	4.27 (0.49)	0.347
Lactate (mmol/L)	Mean (SD)	2.16 (1.22)	2.26 (1.44)	0.025	2.23 (1.13)	2.39 (1.46)	0.096
Hemoglobin (g/dL)	Mean (SD)	9.12 (1.08)	8.98 (1.01)	<0.001	8.85 (0.90)	8.79 (1.02)	0.428
PLT (K/uL)	Mean (SD)	159.45 (71.08)	154.02 (64.05)	0.036	144.31 (71.92)	144.52 (73.09)	0.971
Urea Nitrogen (mg/dL)	Mean (SD)	38.60 (15.71)	39.37 (16.49)	0.184	44.17 (17.69)	46.28 (19.88)	0.141
Creatinine (mg/dL)	Mean (SD)	2.16 (1.09)	2.25 (1.24)	0.037	3.10 (1.31)	3.22 (1.22)	0.236
Outcomes	90-day mortality, *n* (%)	1,313 (19.4)	318 (38.5)	<0.001	163 (22.1)	70 (34.0)	0.001
	30-day mortality, *n* (%)	1,249 (18.4)	301 (36.5)	<0.001	156 (21.2)	65 (31.6)	0.003
	Los, Mean (SD)	4.63 (5.60)	8.65 (9.57)	<0.001	4.17 (4.33)	8.29 (8.14)	<0.001

Continuous variables are presented as means (SD), while categorical variables are presented as counts and percentages. Charlson comorbidity index (CCI) is a tool used to assess the burden of chronic diseases and comorbidities in patients. HR heart rate, RR respiratory rate, SBP systolic blood pressure, MBP mean arterial pressure, DBP diastolic blood pressure, SpO2 pulse blood oxygen saturation, SOFA sequential organ failure Assessment, SAPS II simplified acute physiology score II, GCS Glasgow Coma Scale, PLT platelet, and Los length of stay.

At the initial point of eligibility, patient baseline characteristics were stratified according to the implementation of the RRT strategy. Notably, patients who embarked on the RRT strategy exhibited a higher likelihood of requiring mechanical ventilation [2,532 (37.3) vs. 459 (55.6), *p* < 0.001], using vasopressors [131 (1.9) vs. 33 (4.0), *p* < 0.001], and being diagnosed with sepsis [4,086 (60.2) vs. 631 (76.5), *p* < 0.001]. Moreover, this patient group exhibited a heightened prevalence of comorbidities, as reflected by the Charlson comorbidity index scores [6.83 (3.00) vs. 7.20 (2.92), *p* = 0.001]. The RRT-initiated group also presented higher scores on the SOFA scale [7.32 (3.86) vs. 11.98 (4.29), *p* < 0.001] and the simplified acute physiology score (SAPS) II scale [43.08 (14.50) vs. 54.39 (15.63), *p* < 0.001], indicative of a more severe clinical state. Additionally, we observed modestly elevated lactate levels [2.16 (1.22) vs. 2.26 (1.44), *p* = 0.025] and creatinine levels [2.16 (1.09) vs. 2.25 (1.24), *p* = 0.037] in the RRT-initiated group, along with reduced hemoglobin levels [9.12 (1.08) vs. 8.98 (1.01), *p* < 0.001] and a slightly lower mean platelet count [159.45 (71.08) vs. 154.02 (64.05), *p* = 0.036] compared to the non-RRT group. These disparities were statistically significant, suggesting that patients who initiated RRT were confronting more critical pathophysiological challenges.

Considering the mortality outcomes, 318 patients (38.5%) within the RRT-initiated group died within the 90-day unadjusted period. In contrast, a significantly lower proportion, 19.4% of the 1,313 patients who did not initiate RRT, met the same fate within the 90-day period (*p* < 0.001). A similar pattern emerged when examining the 30-day mortality rate, with 301 patients (36.5%) in the RRT-initiated group and 1,249 patients (18.4%) in the non-RRT group deceased by the 30-day mark (*p* < 0.001). Furthermore, patients who initiated RRT experienced extended hospital stays, with a statistically significant mean difference [4.63 (5.60) vs. 8.65 (9.57), *p* < 0.001] (Table 1). This extended duration could be indicative of the more severe medical conditions that necessitate RRT, thus requiring more intensive hospitalization and surveillance.

SMDs were calculated for all baseline covariates before and after inverse probability weighting. Prior to weighting, several variables (e.g., mechanical ventilation, vasopressor use, sepsis, SOFA score) showed significant imbalance between early and delayed RRT groups, with SMDs > 0.1. After applying IPW, all baseline covariates achieved good balance across both target trials, indicating successful adjustment for confounding. Detailed SMD values are provided in Supplementary Table 3.

### 3.2 Estimates using MIMIC-IV data

#### 3.2.1 Target trial 1

**Figure 2:**
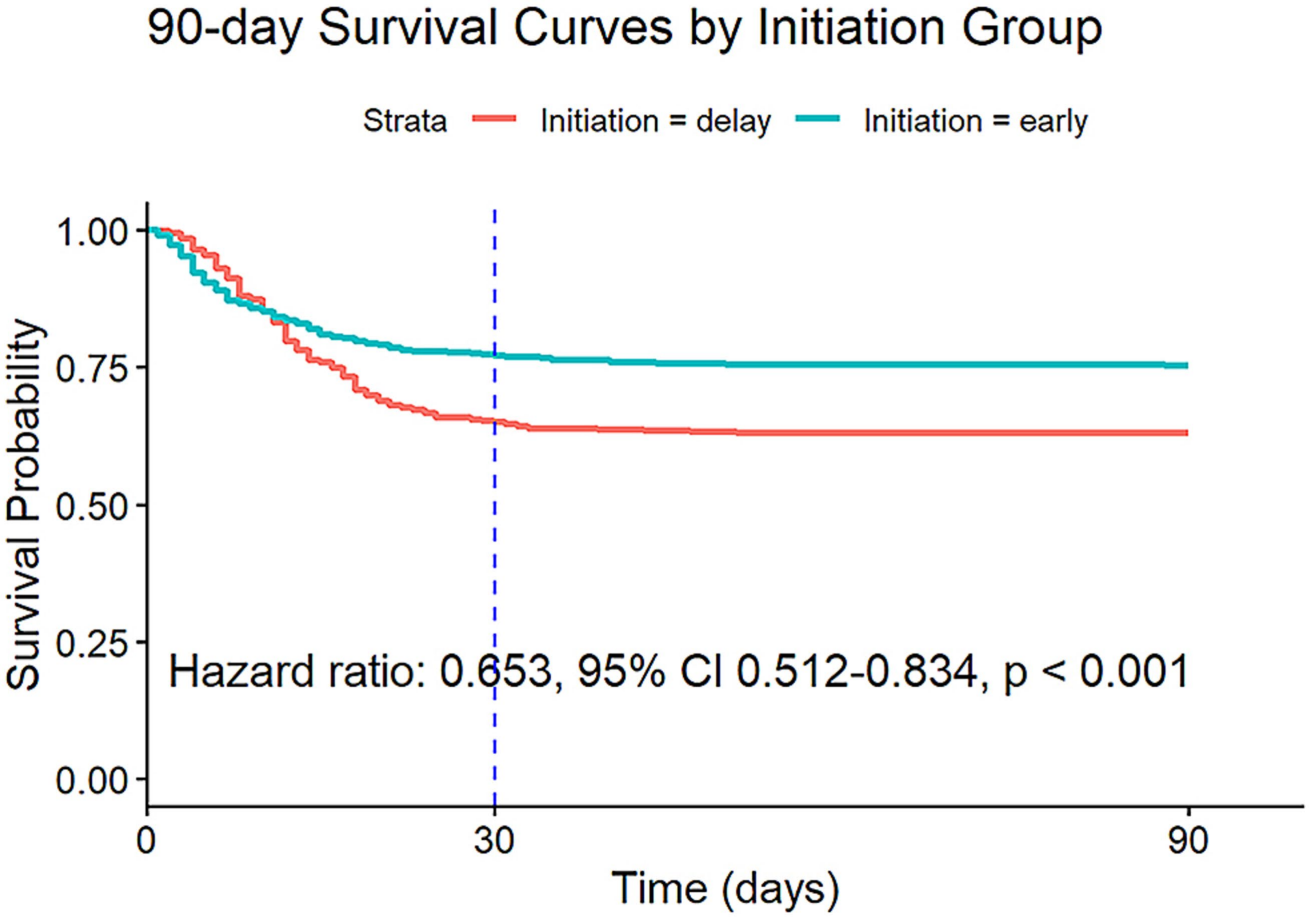
Survival curves estimated from the weighted Cox model (after IPW) for 90-day risk of death for target trial 1, with the dashed line indicating 30 days after the initiation. IPW: inverse probability weighting.

**Figure 3:**
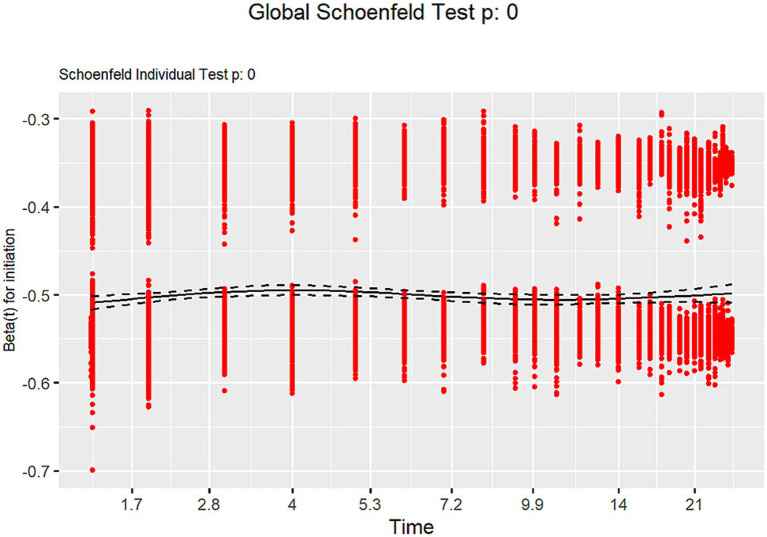
Schoenfeld plot.

The findings of a Cox proportional hazards model with IPW suggest that early initiation of RRT significantly reduced the 90-day mortality risk (HR: 0.653, 95% CI: 0.512–0.834, *p* < 0.001) (Figure 2). Diagnostic analyses using Schoenfeld residuals indicated a violation of the proportional hazards assumption (global Schoenfeld test, *p* < 0.001), potentially limiting the model’s applicability for all covariates (Figure 3). Notwithstanding this limitation, the same Cox model was employed to evaluate the impact on 30-day mortality, which also showed a significant reduction in mortality risk (HR: 0.649, 95% CI: 0.504–0.835, *p* < 0.001) (Supplementary Figure 1).

#### 3.2.2 Target trial 2

**Figure 4:**
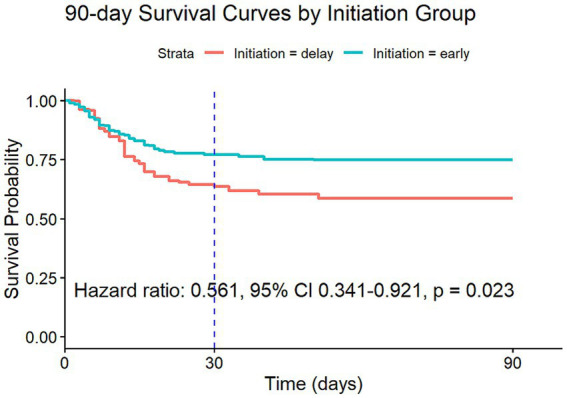
Survival curves estimated from the weighted Cox model (after IPW) for 90-day risk of death for target trial 2.

In the cohort of patients with severe AKI (defined as AKI stage 3 according to KDIGO criteria), 943 patients met the eligibility criteria, of whom 206 (21.8%) initiated RRT within 72 h. Using a Cox proportional hazards model with IPW to account for potential confounding factors, the analysis revealed a significantly lower 90-day mortality risk in this cohort, with an HR of 0.561 (95% CI: 0.341–0.921, *p* = 0.023). Similarly, the 30-day mortality risk was also significantly reduced, with an HR of 0.604 (95% CI: 0.357–1.022, *p* = 0.061). However, the result of the 30-day mortality study was not statistically significant, as detailed in Figure 4, Supplementary Figure 2, and Table 1.

### 3.3 Sensitivity analysis

#### 3.3.1 Target trial 1

Given the significant violation of the PH assumption, primarily driven by the presence of multiple time-dependent covariates, we employed an AFT model as an alternative analytical approach. The AFT model, utilizing a Weibull distribution to fit survival times, confirmed that early initiation of RRT resulted in a 14.8% (95% CI: 14.2–15.4%) reduction in 90-day survival time (95% CI: 14.2–15.4%, *p* < 0.001) and a 14.1% (95% CI: 13.5–14.7%, *p* < 0.001) reduction in 30-day survival time. These findings are summarized in Table 2.

**Table 2:** Results of using AFT models in the target trial 1 and 2 populations.

Group	90-day survival	30-day survival
Target trial 1	14.8% (95% CI: 14.2–15.4%, *p* < 0.001)	14.1% (95% CI: 13.5–14.7%, *p* < 0.001)
Target trial 2	3.08 (95% CI: 3.04–3.13, *p* < 0.001)	2.94 (95% CI: 2.88–2.99, *p* < 0.001)

AFT: Accelerated failure time.

**Figure 5:**
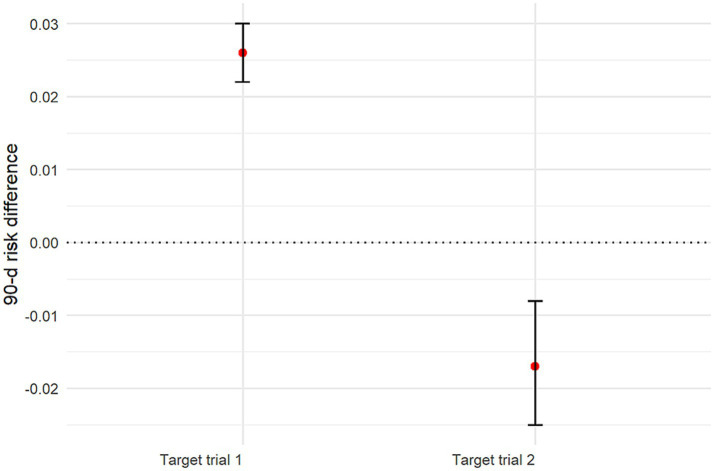
Graph showing estimated 90-day mortality risk differences and 95% CIs for all target trials. CI: Confidence interval.

**Figure 6:**
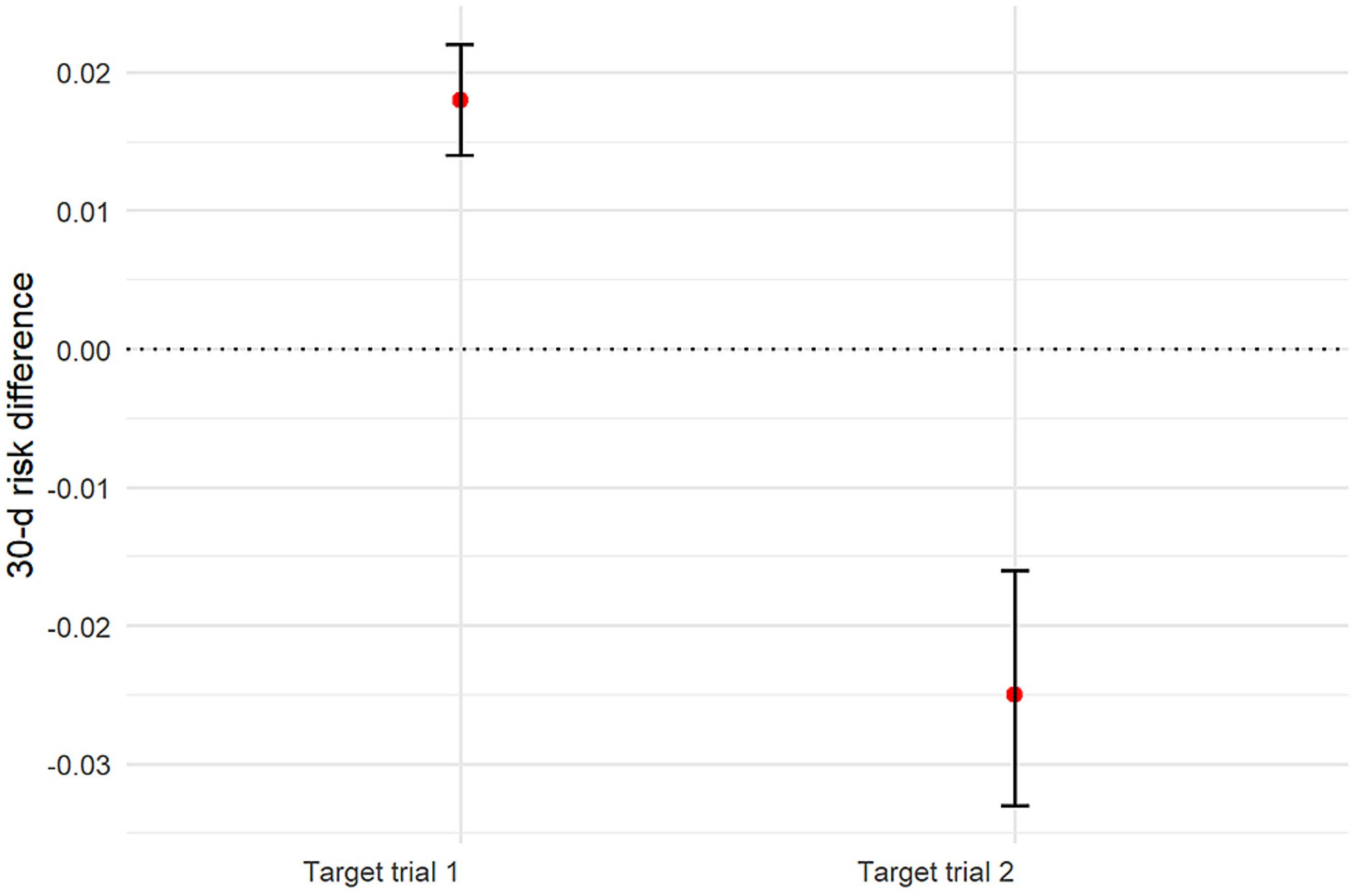
Graph showing estimated 30-day mortality risk differences and 95% CIs for all target trials.

Using sensitivity analyses, we estimated the 90-day risk of death with each treatment strategy by applying the AIPW algorithm. The estimated risk of death at 90 days was 24.5% (95% CI: 24.1–24.9%) with the early initiation strategy of target trial 1 and 21.9% (95% CI: 21.8–22.1%) with the delayed initiation strategy. The risk difference was 2.6 percentage points (95% CI: 2.2–3.0) (Supplementary Figure 3; Figure 5; Table 3). The estimated risk of death at 30 days was 22.7% (95% CI: 22.3–23.1%) with the early initiation strategy of target trial 1 and 20.9% (95% CI: 20.7–21.0%) with the delayed initiation strategy. The risk difference was 1.8 percentage points (95% CI: 1.4–2.2) (Figure 6; Table 4).

**Table 3:** Results for 90-day mortality risk by using AIPW models in the target trial 1 and 2 populations.

Group	Early initiation	Delayed initiation	Risk difference (%)
Target trial 1	24.5% (95% CI: 24.1–24.9%)	21.9% (95% CI: 21.8–22.1%)	2.6 (95% CI: 2.2–3.0)
Target trial 2	25.1% (95% CI: 24.4–25.9%)	26.8% (95% CI: 26.3–27.2%)	−1.7 (95% CI: −2.5 – −0.8)

AIPW: Augmented inverse probability weighting.

**Table 4:** Results for 30-day mortality risk by using AIPW models in the target trial 1 and 2 populations.

Group	Early initiation	Delayed initiation	Risk difference (%)
Target trial 1	22.7% (95% CI: 22.3–23.1%)	20.9% (95% CI: 20.7–21.0%)	1.8 (95% CI: 1.4–2.2)
Target trial 2	23.2% (95% CI: 22.5–24.0%)	25.7% (95% CI: 25.3–26.1%)	−2.5 (95% CI: −3.3 – −1.6)

VIFs were computed for all covariates used in the IPW and outcome models. Across both target trials, all VIF values were below 2, indicating no evidence of significant multicollinearity (Supplementary Table 4).

#### 3.3.2 Target trial 2

In this cohort, the AFT model suggested that 90-day survival was 3.08 times (95% CI: 3.04–3.13, *p* < 0.001) longer and 30-day survival was 2.94 times (95% CI: 2.88–2.99, *p* < 0.001) longer with early initiation of RRT than with delayed initiation of RRT (Table 2).

When focusing on patients with severe AKI, the AIPW analysis yielded a 25.1% (95% CI: 24.4–25.9%) risk of death at 90 days with the early initiation strategy of target trial 2 and 26.8% (95% CI: 26.3–27.2%) with the delayed initiation strategy. The risk difference was −1.7 percentage points (95% CI: −2.5 – −0.8) (Figure 5; Supplementary Figure 4; Table 3). The estimated risk of death at 30 days was 23.2% (95% CI: 22.5–24.0%) with the early initiation strategy and 25.7% (95% CI: 25.3–26.1%) with the delayed initiation strategy. The risk difference was −2.5 percentage points (95% CI: −3.3– −1.6) (Figure 6; Table 4). These refined estimates underscore the robustness of the observed associations and provide a nuanced perspective on the temporal trends in mortality risks among patients with severe AKI.

## 4 Discussion

Our study employs target trial emulation methods, leveraging causal inference techniques and data from the MIMIC-IV database, to re-examine the critical clinical question of whether early initiation of RRT improves outcomes in critically ill patients with AKI. Methodologically, we adopted a two-stage analytical strategy. Target trial 1 (exploratory analysis in AKI stage ≥1) aimed to assess the broader hypothesis-generating question of RRT timing thresholds in mild-to-severe AKI, while target trial 2 (primary analysis in stage 3 AKI) focused on resolving the equipoise in severe AKI management. Our findings yielded robust and distinct conclusions that challenged some previous RCTs, underscoring the importance of further research in this area (6, 41). Specifically, this study demonstrates that early RRT initiation is associated with significantly lower 90- and 30-day mortality rates compared to delayed initiation, particularly in patients with severe AKI (stage 3 or above); however, it does not show prognostic benefits and could be associated with worse outcomes in the broader AKI population (stage ≥1).

Methodologically, the study highlights key differences in analytical approaches, wherein traditional Cox regression analysis with IPW initially suggested benefits of early RRT initiation. However, this finding was not aligned with AFT models, which showed reductions in survival time with early RRT in the broader AKI cohort. This discrepancy underscores the importance of using advanced statistical methods, such as AFT and AIPW, to account for time-varying confounding and accurately capture the impact of treatment timing. The prioritization of stage 3 AKI in our primary analysis aligns with three key clinical realities: (1) Severe AKI patients exhibit distinct pathophysiological trajectories requiring urgent intervention; (2) Current guidelines lack consensus on RRT timing thresholds precisely in this high-risk population; (3) Heterogeneous practice patterns in milder AKI stages (1–2) introduce significant confounding, which our restricted cohort design mitigates.

Overall, the results suggest that early initiation of RRT may have potential benefits in the management of severe AKI (stage 3) in intensive care settings, although the evidence is not yet definitive. Further research is needed to clarify the optimal timing for RRT initiation in different patient populations. However, in less severe AKI cases, early RRT may not be beneficial and could potentially worsen outcomes. These findings suggest that clinicians should adopt a personalized approach by carefully considering the severity of AKI and individual patient characteristics when deciding on the timing of RRT initiation to optimize patient outcomes.

Our findings in severe AKI (stage 3) carry particular clinical relevance. By excluding patients with transient or self-limited AKI (predominantly stage 1–2), target trial 2 isolates a population where RRT initiation decisions are both clinically urgent and mechanistically plausible. This design choice addresses a major limitation of prior RCTs that include heterogeneous AKI populations, potentially obscuring treatment effects.

Our findings contrast with several previous RCTs investigating the impact of RRT timing on mortality outcomes in patients with AKI. The ELAIN trial demonstrated the benefits of early RRT initiation in surgical AKI patients (13), while the AKIKI trial did not observe such advantages, suggesting that early RRT might not be necessary in some cases (19). Similarly, the IDEAL-ICU trial found no difference in the 90-day mortality between early and delayed RRT initiation (10), questioning the routine application of early RRT in septic AKI patients.

While the mortality rate in our severe AKI cohort appears lower than some historical cohorts, the risk difference (−1.7%) between early and delayed RRT remains clinically meaningful. This finding is consistent with the single-center ELAIN trial, which demonstrated improved survival with early RRT initiation in severe AKI.

The differences in prognosis between this study and others in the overall AKI patient cohort can be attributed to several factors. First, previous studies using Cox models did not incorporate time-dependent covariates and treatment measures, or if they did, could have violated the proportional hazards assumption (42), potentially leading to estimation bias in heterogeneous populations, such as the overall AKI cohort. In contrast, our study employed IPW and AIPW to account for time-dependent confounding variables (35, 36), providing a more robust estimation of treatment effects. Additionally, the overall AKI cohort in previous studies may have included milder cases (stage 2), where early RRT initiation might offer no benefit or even be harmful due to the inherent risks of the treatment itself. In contrast, in severe AKI (stage 3), the benefits of RRT may outweigh the risks, as demonstrated in our study.

Second, the definition and staging of AKI may vary across studies, leading to heterogeneity in patient populations. In the IDEAL-ICU study, AKI was classified using the RIFLE criteria (43), which may not be directly comparable to other studies using different criteria, such as the KDIGO criteria used in our study. This variability in definitions could result in differences in AKI severity across studies, thereby influencing outcomes.

Third, the patient population in the IDEAL-ICU study was specifically limited to those with early septic shock, a highly heterogeneous and complex condition associated with high mortality. Sepsis itself is a strong independent predictor of mortality, and the interaction between sepsis and AKI could contribute to worse outcomes in this subset of patients. In contrast, our study included a more diverse population of AKI patients, including those with severe AKI (stage 3), which could have different treatment implications.

Furthermore, the timing and mode of RRT initiation could influence outcomes. In the IDEAL-ICU study (44), RRT was initiated early in the intervention group. However, the benefits of early RRT could be offset by the risks associated with the procedure, such as bleeding complications due to anticoagulation or the pro-inflammatory effects of the dialysis circuit (45, 46). Additionally, the use of regional citrate anticoagulation could have specific effects on biocompatibility and outcomes that differ from other anticoagulation strategies used in previous trials.

Finally, methodological differences, such as sample size, follow-up duration, and handling of censored data, could also explain the discrepancies in results. The IDEAL-ICU study had a relatively large sample size. However, the trial was stopped early due to futility, which could have limited the power to detect differences in mortality between groups. Additionally, the high rate of censored data (patients who did not require RRT or died before RRT initiation) in the delayed group could introduce bias, as these patients could have had a different prognosis compared to those who ultimately required RRT.

In summary, our study provides robust evidence supporting the early initiation of RRT in critically ill patients with severe AKI (stage 3), while suggesting that early RRT may not be beneficial in milder cases. These findings highlight the importance of personalized treatment decisions based on AKI severity and suggest that future trials should focus on specific subgroups of AKI patients to refine treatment guidelines.

A significant strength of our research lies in the integration of observational data from real-world patient cohorts, offering a precise reflection of the diverse manifestations of AKI in clinical practice. By employing target trial emulation, we leveraged real-world methodologies to enhance the relevance and applicability of our findings. This approach, utilizing doubly robust estimation and machine learning techniques, not only replicates benchmark results from randomized clinical trials but also uncovers nuanced insights, such as the potential harms associated with early RRT in patients with less severe AKI. In contrast to traditional Cox regression analysis, target trial emulation provides a more accurate and reliable method of statistical inference, particularly in evaluating the impact of early versus delayed RRT on mortality (21). Through IPW, we effectively balanced treatment groups based on measured covariates, thereby enhancing the robustness of our causal inferences. Sensitivity analyses confirmed the consistency of our findings, reinforcing the reliability of our conclusions.

This study underscores the critical importance of patient selection and the timing of intervention in the management of AKI. It highlights that early initiation of RRT is beneficial in patients with severe AKI, contrary to those with less severe AKI. The inclusion of severe AKI patients or early interventions in previous studies might have obscured the full potential benefits of RRT, consistent with earlier findings. This underscores the need for more precise patient selection and timely intervention, warranting further RCTs to confirm these observations.

### 4.1 Limitations

Several limitations should be considered when interpreting the results of this study. First, the reliance on data solely from the single-center MIMIC-IV database represents a significant limitation to the generalizability of our findings. Patient demographics, clinical practices (such as thresholds for RRT initiation), and resource availability (including access to specialized care and socioeconomic factors) can vary substantially across different healthcare institutions and geographic regions. Consequently, the patient population and management strategies captured in this single-center dataset may not fully reflect the broader spectrum of critically ill patients with AKI, potentially limiting the external validity of our conclusions. To enhance the robustness and generalizability of future research, incorporating multi-center data would be highly valuable.

Second, despite our efforts to adjust for confounding factors, the possibility of residual confounding by unmeasured variables remains. Unmeasured variables, such as specific comorbidities or treatment practices that vary between centers, could have influenced the observed outcomes, introducing potential bias into our analysis. Although we adjusted for measured confounders, unmeasured factors such as clinician preference for RRT timing or variations in supportive care protocols could further influence outcomes.

In addition, while target trial emulation was employed to mitigate biases, the potential for residual biases, such as immortal time bias, cannot be entirely ruled out. Despite these limitations, our findings provide valuable insights and suggest promising directions for the management of severe AKI. However, we acknowledge the need for cautious interpretation and emphasize the importance of replicating our study in diverse, larger populations to confirm our results.

We acknowledge that early RRT initiation in KDIGO stage 1 AKI is uncommon in clinical practice. However, the inclusion of stage 1 AKI in target trial 1 was intentional to address the guideline gap regarding timing thresholds, even in mild renal impairment. This exploratory analysis revealed potential harms of early RRT in the broader AKI cohort (stage ≥1), reinforcing the need for severity-stratified approaches. Conversely, in target trial 2 (stage 3 AKI), early RRT initiation was associated with significantly lower 90-day and 30-day mortality rates compared to delayed initiation. This dichotomy highlights the critical importance of differentiating therapeutic strategies based on AKI severity.

### 4.2 Future research directions

To address the limitations of our study and the complexities surrounding the optimal timing for RRT initiation in AKI, future research should prioritize large-scale, multi-center trials to validate and generalize our findings. These trials should incorporate diverse patient populations and explore various RRT modalities to develop more nuanced guidelines tailored to different clinical scenarios and AKI severities. Specifically, stratifying patients based on AKI stage and other relevant factors could enhance the precision of these guidelines.

Moreover, identifying biomarkers that predict which patients are most likely to benefit from early RRT is a critical area for future investigation. Exploring specific biomarkers, such as inflammatory markers or kidney injury molecules, could enable personalized treatment plans. Additionally, advances in precision medicine and machine learning algorithms offer promising avenues for developing predictive models that guide RRT initiation decisions, thereby optimizing patient outcomes.

Finally, future studies should also consider the practical aspects of implementing early RRT in clinical settings, including resource requirements and feasibility across different healthcare environments. By addressing these areas, we can further refine the management of AKI and improve patient care.

## 5 Conclusion

In summary, this study provides robust evidence supporting the early initiation of RRT in critically ill patients with AKI, particularly in critically ill patients (stage 3 or above), as it significantly reduces 90-day mortality in patients. However, the findings suggest that in less severe patients, early RRT may not be beneficial and could potentially worsen outcomes. These results highlight the importance of personalized treatment decisions, whereby the timing and necessity of RRT should be carefully considered according to the severity of AKI.

The study’s methodological strengths, including the use of target trial emulation and advanced statistical techniques, enhance the precision of these estimates and provide valuable insights for clinicians. These findings contribute to the growing body of evidence that suggests a nuanced approach to AKI management, moving away from a one-size-fits-all strategy.

Our findings complement existing RCTs by addressing key limitations through real-world data and causal inference methods. While prior RCTs like AKIKI and STARRT-AKI established foundational evidence, their strict eligibility criteria excluded high-risk subgroups, such as elderly patients with multi-organ failure, and did not stratify outcomes by AKI severity. By emulating a target trial in a heterogeneous ICU population, we identified a critical dichotomy, which is, early RRT reduced mortality in severe AKI (stage 3, HR: 0.561) but increased harm in milder cases (stage ≥1, risk difference 2.6%). This underscores the need for precision RCTs targeting specific subgroups (stage 3 AKI with hyperinflammatory biomarkers) rather than broad populations. Future trials should integrate biomarker-guided thresholds such as urinary [TIMP-2]·[IGFBP7] to personalize RRT timing, thereby bridging the gap between RCT homogeneity and real-world complexity.

## Data Availability

The original contributions presented in the study are included in the article/Supplementary material. Further inquiries can be directed to the corresponding authors.
