# Supplementary material for: The early bird in renal rescue: timing matters in acute kidney injury management—insights from target trial emulation
Source: Front Med (Lausanne). 2025 Sep 4;12:1645046. doi: 10.3389/fmed.2025.1645046 (PMC12443691; doi:10.3389/fmed.2025.1645046)

**Supplemental Materials for “The Early Bird in Renal Rescue: Timing Matters in AKI Management—Insights from Target Trial Emulation”**

**Supplementary Methods**

**1. Data Structure and Processing**

When constructing this dataset, we executed SQL queries on MIMIC-IV through Navicat Premium 16, resulting in a long-format data frame where each row represents an hourly interval since ICU admission. Consequently, each subject has multiple observation points (up to 120 hours), including physiological data and RRT status information. Physiological data is summarized as the mean within each hourly interval, and exposure information is represented as Yes/No. In a new hourly interval, if RRT is set to 1 (Yes), it means that the treatment has begun, but it is not possible to know the exact time of occurrence within that hour. This is a potential issue because physiological data may be collected after exposure, thus representing post-exposure information. To address this problem, it was decided to advance the physiological information by one hour. While this method allows researchers to confidently state that the physiological data indeed represents the state before initiating RRT, it results in the deletion of the first hour of ICU observation for all patients. To overcome this problem, it was thus decided to move (lag) the physiological information one hour ahead.

**2. Hourly RRT Probability Estimation**

For the current study, the investigators estimated the hourly probability of patients receiving RRT based on the aforementioned physiological data. This probability was calculated for each subject hourly until the end of the 72-hour period or until the patient presented any exclusion criteria, no longer met the inclusion criteria, died, or left the ICU.

**3. Missing Data Handling**

For time-dependent clinical data, last observation carried forward (LOCF) imputation was used, assuming that most missing data reflected the absence of any meaningful change, as significant deviations are more likely to be recorded. After this, the amount of missing data was minimal, as shown below: For heart rate and respiratory rate, missing data accounted for 2.1% of the observations. Systolic blood pressure data were missing in 7.3% of the observations.

For non-time-dependent clinical data and cases where missing values still existed, multiple imputations were performed using the mice package. For this, each missing value was imputed 25 times, and the median of the imputations was selected. Descriptive analysis was conducted to evaluate whether the distribution of the imputed variables and their correlation with other variables had deviated from the pre-imputation values.

**4. Target trial emulation**

**Cloning:** Each eligible subject was cloned to create 2 replicates, mirroring the process in a clinical trial where each patient could, in theory, receive different treatment strategies under identical initial conditions. These clones were randomly assigned to one of the two predefined treatment strategies at the commencement of the follow-up period (T0), thereby setting the stage for a emulated comparison of treatment effects.

**Censoring:** The study implemented hourly checkpoints to assess the fidelity of the replicates to their assigned treatment strategy. Any deviation from the assigned strategy resulted in the censoring of the respective replicate. This process was critical in maintaining analytical consistency, as it emulated the strict protocol adherence characteristic of well-controlled clinical trials, ensuring that the analysis reflected the intended treatment effects without the confounding influence of protocol violations.

**Weighting:** To correct for potential selection bias introduced by the censoring process, each individual was assigned a time-varying inverse probability weight. These weights were calculated based on the conditional probability of a replicate remaining on its assigned treatment strategy, contingent upon the individual's baseline characteristics. To calculate this, logistic Regression models with the initiation of RRT as the dependent variable and variables supposed to play a role in the decision for initiation were used as independent variables. The variables included the time since fulfilling the inclusion criteria, age, gender, and comorbidities as measured by the Charlson Comorbidity Index (CCI), Glasgow Coma Scale (GCS), Sequential Organ Failure Assessment (SOFA), heart rate, respiratory rate, systolic blood pressure, mean arterial pressure and diastolic blood pressure.

**5. Propensity Score Estimation**

To estimate the propensity score, a logistic regression model was used, with the receipt of RRT as the dependent variable and the following covariates: age, gender, SOFA score, Charlson score, heart rate, respiratory rate, diastolic blood pressure, and oxygen saturation, presence of sepsis, and the use of vasoactive drugs. For each hourly observation, each patient had a probability between 0 and 1 of receiving RRT. These probabilities were later used to estimate stabilized inverse probability weights (IPW). Since this approach carries the risk of inducing weight inflation in outliers, weight truncation was performed at the 99th percentile and above. This allowed the estimates to maintain precision while reducing standard errors.

**6. Survival Analysis**

90-day survival was analyzed using a weighted Cox model (including stabilized IPW weights) and further adjusted for systolic blood pressure, mean arterial pressure, temperature, pH, bicarbonate, lactate, hemoglobin, urea nitrogen, and creatinine. Hazard ratios (HR) are reported as an average treatment effect over the study time, and survival curves were constructed using the estimates from the Cox model. Robust standard errors were estimated to calculate 95% confidence intervals, accounting for the multiplicity of same-subject observations. Additionally, we decided to perform doubly robust estimators: augmented inverse probability weighting (AIPW). Since all participants in our study provided complete data with no missing values, we used logistic regression for the relevant probability estimates, which significantly simplified the computational process. In the exposure model, we used the same variables as described in the estimation of IPW, and we added systolic blood pressure, mean arterial pressure, temperature, pH, bicarbonate, lactate, hemoglobin, urea nitrogen, and creatinine to the outcome model. We ran these analyses using the AIPW package.

**7. Alternative Analysis Method: AFT Model**

The fundamental assumption of the Cox model—that the hazard ratio remains constant over time—needs to be confirmed by verifying the PH assumption. Despite our efforts to minimize bias through the use of weighted Cox models and IPW strategies, test results showed that the PH assumption was not fully confirmed, indicating that traditional Cox models may be insufficient to capture the dynamic changes in hazard ratios over time. Therefore, this study further adopted the accelerated failure time (AFT) model as an alternative analytical method. Unlike traditional Cox proportional hazards models, the AFT model is not constrained by the PH assumption, providing a more flexible framework for describing the relationship between survival time and various outcome variables . The model controls for potential confounders through stratification, thereby offering a more accurate estimate of mortality risk.

Supplemental Table 1. Description of the target trial

Patients were considered eligible for inclusion in the study if they had been admitted to the ICU for the first time, had a stay in the ICU exceeding one day, were over 18 years of age, and had AKI stage > 0 according to the KDIGO criteria. We excluded patients from the Neurological ICU as well as those with pre-existing chronic kidney disease or respiratory rate less than 10 breaths per minute.

|  | **Target trial** | **Emulated trial** |
| --- | --- | --- |
| Population | Inclusion criteria: Patients were considered eligible if they had been admitted to the ICU for the first time, had a stay in the ICU exceeding 72 hours, were over 18 years old, and had AKI stage ≥ 1 according to the KDIGO criteria.  Exclusion criteria: Patients admitted to the Neurological ICU, with pre-existing chronic kidney disease or respiratory rate < 10 bpm. | Inclusion criteria: Patients were considered eligible if they had been admitted to the ICU for the first time, had a stay in the ICU exceeding 72 hours, were over 18 years old, and had AKI stage ≥ 1 according to the KDIGO criteria.  Exclusion criteria: Patients admitted to the Neurological ICU, with pre-existing chronic kidney disease or respiratory rate < 10 bpm. |
| Intervention | Initiation | Initiation within one hour |
| Comparison |  | At baseline, patients who met the criteria for the intervention were assigned to the corresponding group, while those who did not meet the criteria were considered ineligible for the study. |
| Study design | Randomization at the time of enrollment. The RRT strategy should be initiated as soon as possible. If the strategy is not carried out within one hour after being randomly assigned to the initiation group, it is considered a protocol violation. | Patient identification at the time of initial enrollment (target trial 1). Thereafter, if they remain untreated with the RRT strategy and continue to meet all inclusion criteria without any exclusion criteria, they will still be identified as such every hour within 72 hours. |
| Outcome | 90-day mortality risk (hazard ratio)  30-day mortality risk (hazard ratio) | 90-day mortality risk (hazard ratio)  30-day mortality risk (hazard ratio) |
| Causal contrast | Intention to treat | Intention to treat |
| Statistical analysis | Cox model for 90-day and 30-daymortality (hazard ratio).  AFT model for 90-day and 30-day mortality. | In each trial, estimate the probability of receiving the RRT strategy and construct the stable inverse probability weights for treatment.  Weighted Cox model for main outcome. Survival curves derived from stratified (by treatment groups) Cox models.  The weighted AFT for the primary outcome. |

Supplemental Table 2. Study flowchart for each of the 72 hours. At each hour, the number of patients who initiated the RRT strategy and those who did not initiate the RRT strategy is displayed. We used hour 1 as first hour after eligibility.

| Hours since eligibility | No initiation | Initiation |
| --- | --- | --- |
| 1 | 7602 | 5 |
| 2 | 7596 | 4 |
| 3 | 7604 | 1 |
| 4 | 7593 | 7 |
| 5 | 7586 | 12 |
| 6 | 7593 | 7 |
| 7 | 7588 | 6 |
| 8 | 7589 | 8 |
| 9 | 7591 | 4 |
| 10 | 7588 | 4 |
| 11 | 7588 | 8 |
| 12 | 7579 | 11 |
| 13 | 7594 | 4 |
| 14 | 7591 | 6 |
| 15 | 7592 | 4 |
| 16 | 7590 | 3 |
| 17 | 7591 | 6 |
| 18 | 7590 | 5 |
| 19 | 7593 | 5 |
| 20 | 7589 | 4 |
| 21 | 7582 | 6 |
| 22 | 7584 | 7 |
| 23 | 7585 | 11 |
| 24 | 7595 | 5 |
| 25 | 7588 | 10 |
| 26 | 7567 | 5 |
| 27 | 7449 | 3 |
| 28 | 7315 | 6 |
| 29 | 7173 | 5 |
| 30 | 7062 | 14 |
| 31 | 6967 | 6 |
| 32 | 6867 | 2 |
| 33 | 6783 | 2 |
| 34 | 6695 | 6 |
| 35 | 6629 | 3 |
| 36 | 6556 | 1 |
| 37 | 6480 | 0 |
| 38 | 6405 | 0 |
| 39 | 6321 | 4 |
| 40 | 6237 | 2 |
| 41 | 6175 | 2 |
| 42 | 6069 | 1 |
| 43 | 5992 | 4 |
| 44 | 5902 | 2 |
| 45 | 5784 | 1 |
| 46 | 5675 | 7 |
| 47 | 5559 | 2 |
| 48 | 5442 | 2 |
| 49 | 5320 | 2 |
| 50 | 5238 | 4 |
| 51 | 5130 | 3 |
| 52 | 5032 | 5 |
| 53 | 4954 | 1 |
| 54 | 4859 | 2 |
| 55 | 4783 | 1 |
| 56 | 4710 | 1 |
| 57 | 4651 | 1 |
| 58 | 4578 | 3 |
| 59 | 4527 | 1 |
| 60 | 4481 | 1 |
| 61 | 4443 | 1 |
| 62 | 4397 | 0 |
| 63 | 4355 | 0 |
| 64 | 4300 | 0 |
| 65 | 4250 | 2 |
| 66 | 4198 | 1 |
| 67 | 4145 | 0 |
| 68 | 4086 | 0 |
| 69 | 4032 | 0 |
| 70 | 3965 | 3 |
| 71 | 3907 | 0 |
| 72 | 3822 | 2 |

Supplemental Table 3. Standardized Mean Differences (SMD) for Key Covariates Before and After IPW (Target Trial 1)

| Covariate​ | SMD (Before IPW)​ | SMD (After IPW) |
| --- | --- | --- |
| SOFA score | 0.9 | 0.15 |
| Charlson Index | 0.18 | 0.13 |
| Sepsis | 0.25 | 0.06 |
| Vasopressor | 0.66 | 0.12 |
| HR | 0.14 | 0.02 |
| RR | 0.23 | 0.06 |

Supplemental Table 4. Variance Inflation Factor (VIF) for Covariates in the Propensity Score Model

| Variable | VIF |
| --- | --- |
| Age | 1.52 |
| Gender | 1.02 |
| SOFA | 1.25 |
| Charlson | 1.46 |
| HR | 1.15 |
| RR | 1.08 |
| DBP | 1.10 |
| SpO2 | 1.02 |
| Sepsis | 1.12 |
| Vasopressor | 1.11 |

Supplemental Figure 1. Survival curves estimated from the weighted Cox model (after IPW) for 30-day risk of death for target trial 1.

**
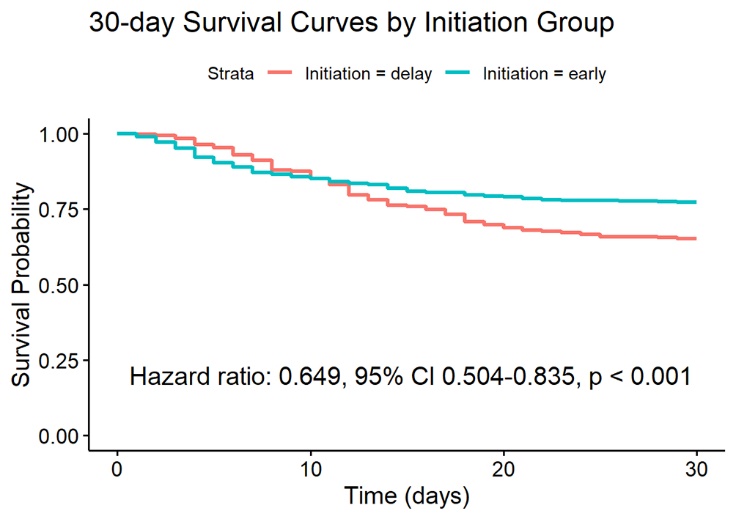
**

Supplemental Figure 2. Survival curves estimated from the weighted Cox model (after IPW) for 30-day risk of death for target trial 2.

**
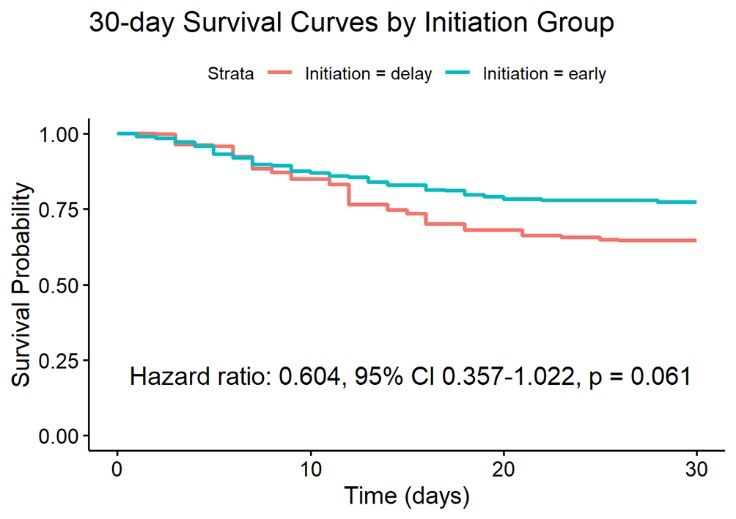
**

Supplemental Figure 3. Propensity score distribution by groups of treatment (target trial 1).

**
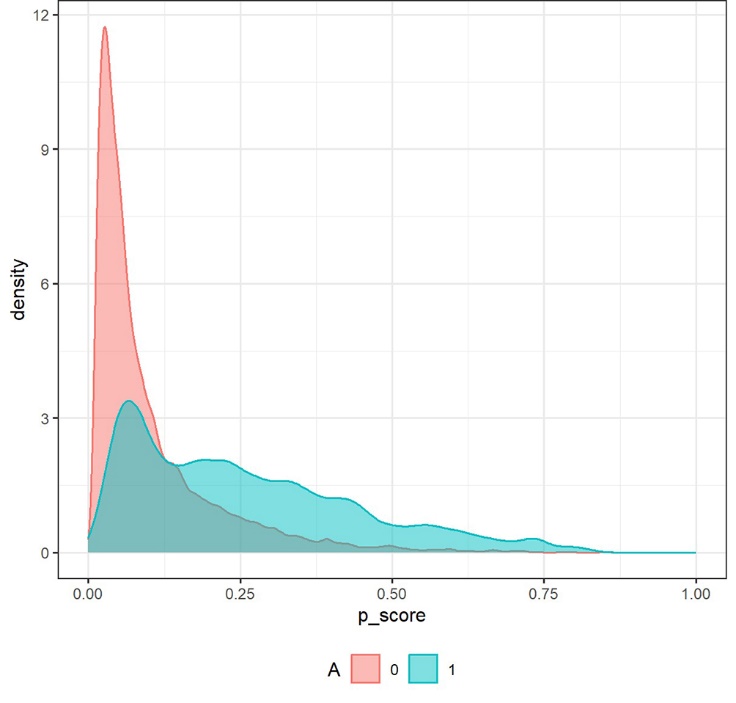
**

Supplemental Figure 4. Propensity score distribution by groups of treatment (target trial 2).

**
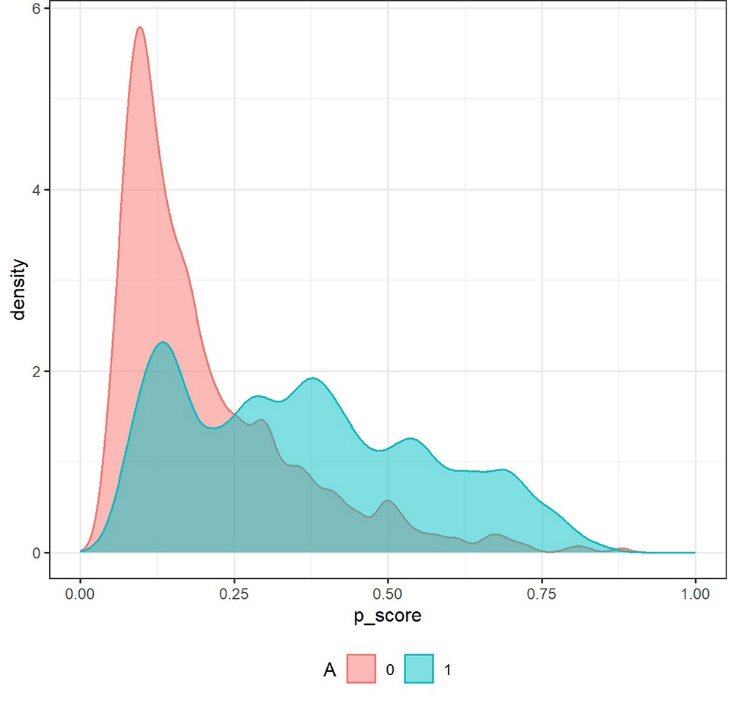
**

Supplemental Figure 5. Early initiation defined as RRT started within 6 hours of diagnosis. Survival curves estimated from the weighted Cox model (after IPW) for 90-day risk of death for target trial 1, with the dashed line indicating 30 days after the initial.


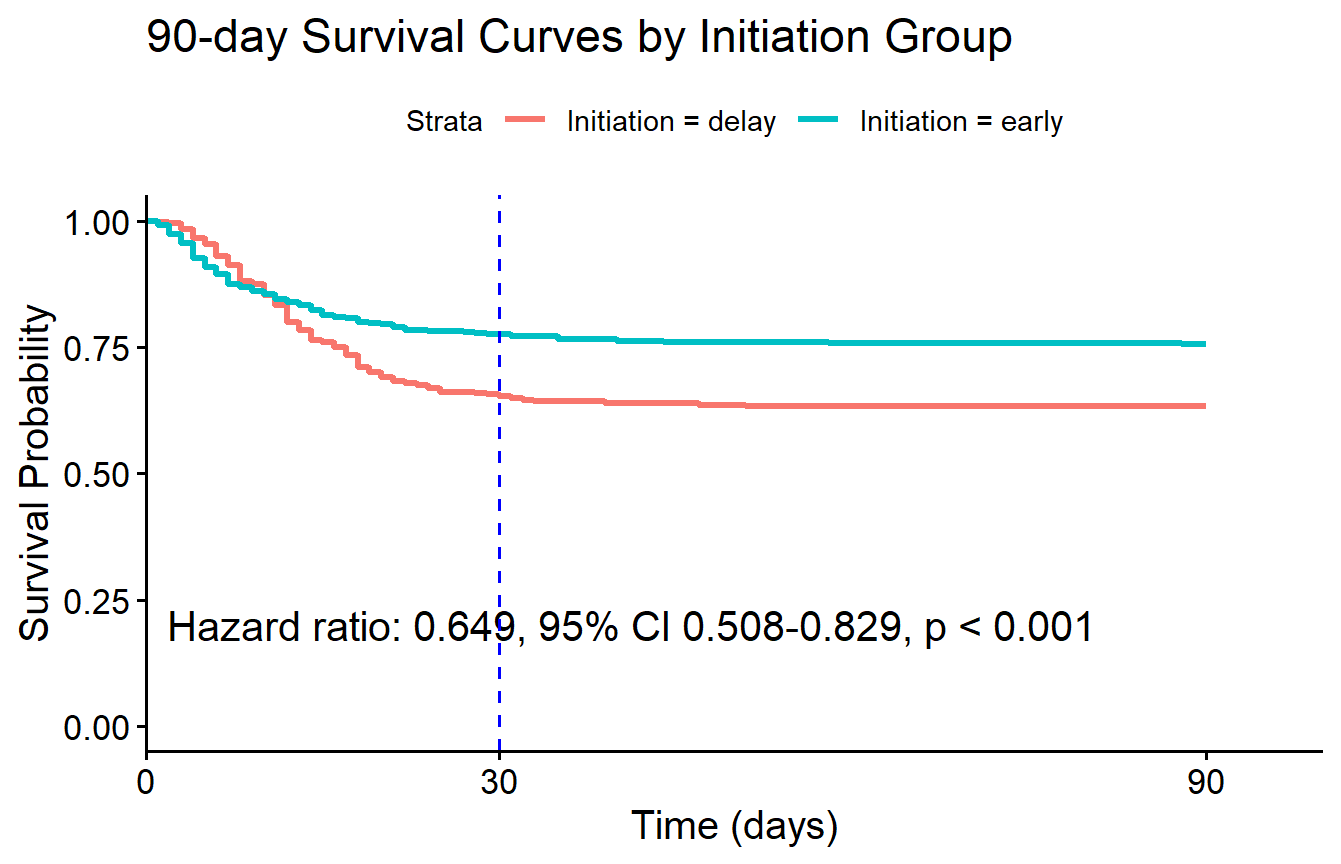


Supplementary Figure 6: Study Flowchart


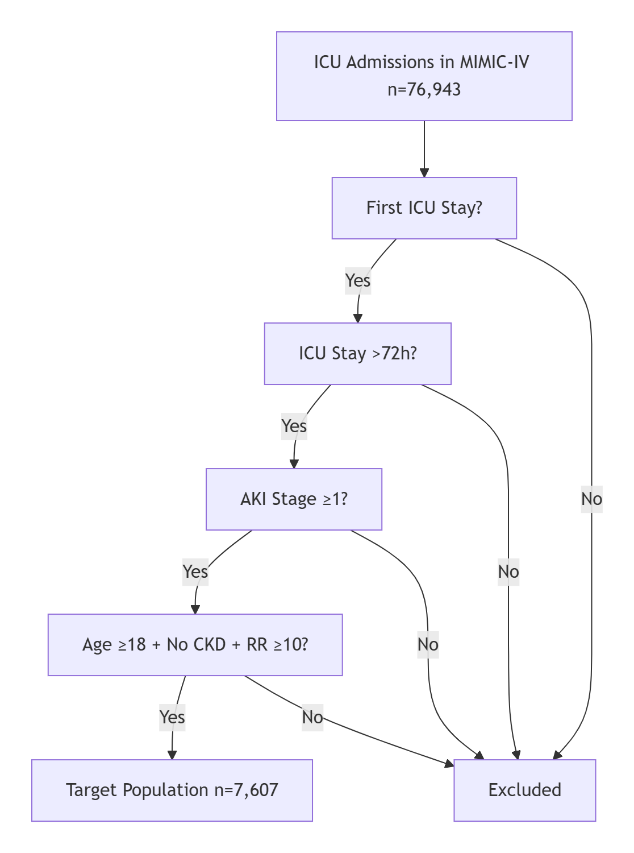


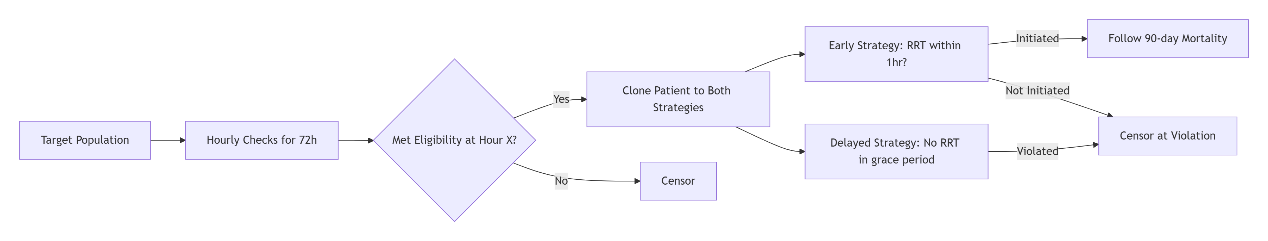

Supplement: Supplementary file 1 [file Data_Sheet_1.docx]
